# Origins of All-Optical Generation of Plasmons in Graphene

**DOI:** 10.1038/s41598-019-39961-1

**Published:** 2019-03-01

**Authors:** C. J. Tollerton, J. Bohn, T. J. Constant, S. A. R. Horsley, D. E. Chang, E. Hendry, D. Z. Li

**Affiliations:** 1 Department of Physics and Astronomy, University of Exeter, Exeter, UK; 2 ICFO-Institut de Ciencies Fotoniques, The Barcelona Institute of Science and Technology, 08860 Castelldefels, Barcelona Spain; 3 ICREA-Institució Catalana de Recerca i Estudis Avançats, 08015 Barcleona, Spain

## Abstract

Graphene, despite its centrosymmetric structure, is predicted to have a substantial second order nonlinearity, arising from non-local effects. However, there is disagreement between several published theories and experimental data. Here we derive an expression for the second order conductivity of graphene in the non-local regime using perturbation theory, concentrating on the difference frequency mixing process, and compare our results with those already published. We find a second-order conductivity (σ^(2)^ ≈ 10^−17^ AmV^−2^) that is approximately three orders of magnitude less than that estimated from recent experimental results. This indicates that nonlinear optical coupling to plasmons in graphene cannot be described perturbatively through the electronic nonlinearity, as previously thought. We also show that this discrepancy cannot be attributed to the bulk optical nonlinearity of the substrate. As a possible alternative, we present a simple theoretical model of how a non-linearity can arise from photothermal effects, which generates a field at least two orders of magnitude larger than that found from perturbation theory.

## Introduction

Graphene, with its linear dispersion and a linear density of states for electrons^1^, exhibits remarkable optical properties such as universal, linear optical conductivity^2^. Moreover, while a single layer of graphene is relatively transparent (due to its mono-layer thickness), the nonlinear optical conductivity has been shown to be surprisingly large^3,4^. This is particularly true for the second order nonlinearity, which is unexpected within the dipole approximation for a centrosymmetric material^5^, but can be substantial in graphene due to non-locality^6–11^.

The 2D nature of graphene also gives rise to plasmons with wavelengths that are substantially smaller than free-space electromagnetic radiation of the same frequency by approximately two orders of magnitude^12^, generating large non-local effects. Of particular interest here are the nonlinearities in the infrared spectral region, which may be enhanced due to the presence of plasmons^12,13^. Recently, B. Yao^7^ and Constant^14^ have independently reported experimental measurements of a frequency mixing process, with a difference frequency generation (DFG) in the mid-infrared, that implied enhancement due to the presence of plasmons. Such an all-optical coupling scheme for plasmon generation in graphene holds great promise, for example, in the design of plasmon sensors or new THz sources.

Given the large nonlinearities observed in graphene and its potential for optical devices, it would be highly beneficial to develop a quantitative, microscopic understanding of its origin. There already exist a number of calculations in literature of the second-order conductivity in graphene for DFG conditions, in a regime where the incident fields are assumed to only weakly perturb the equilibrium Fermi carrier distribution. However, the conclusions are not all consistent with one another, and differing models or assumptions have been used to point to consistency with experimental results. Here, our primary objectives are to show definitively that the correct perturbative model of graphene nonlinearities does not describe well existing experiments, and to propose an alternative non-perturbative mechanism based upon photothermal effects, whose predicted strength is closer to experimental values. First, we obtain a second-order conductivity of graphene for DFG that is different from the first theoretical calculation specifically for DFG^6^, but provides confirmation to a set of other published theoretical results^8,9,11^. While the final result in itself is not new, we derive it in a different fashion based upon the Peierls substitution^15^, which avoids issues that arise in the calculation of the linear conductivity using a vector potential with the Dirac Hamiltonian^11^. We then present a set of original results, beginning with an application of this theoretical result to the experimental conditions of Constant^14^ and a quantitative comparison between theory and experiment highlighting a large discrepancy. We explicitly show that the experimental effects observed by Constant^14^ and B. Yao^7^ cannot be attributed to the non-linearity of the substrate. Finally, we discuss other possible contributions to the wave mixing signals observed. In particular, we derive a model showing how such a signal could arise from photothermal effects, and estimate a difference frequency field that is two orders of magnitude larger than that from perturbation theory.

## Results and Discussion

### Perturbation theory

Formally, the interaction of an electron with a vector potential $\overrightarrow{A}$ can be incorporated into a Hamiltonian via the substitution $\hat{\overrightarrow{p}}\to \hat{\overrightarrow{p}}+e\overrightarrow{A}$, where $\hat{\overrightarrow{p}}$ is the canonical momentum and *e* is the elemental charge. Absent the vector potential, an electron in a periodic crystal potential $V(\overrightarrow{r})$, with Hamiltonian $\hat{H}={\hat{\overrightarrow{p}}}^{2}/2m+V(\overrightarrow{r})$, where *m* is the bare mass of an electron, can be formally diagonalized to produce a band structure. In the case of graphene, the Hamiltonian is typically taken to be of a tight-binding form. The Peierls substitution^15^ formally enables one to incorporate the vector potential into such a postulated model, avoiding the need to actually solve for the eigenstates of the Hamiltonian with the replacement $\hat{\overrightarrow{p}}\to \hat{\overrightarrow{p}}+e\overrightarrow{A}$. The tight-binding Hamiltonian of graphene with $\overrightarrow{A}$ thus reads:

1 $$\hat{H}={\mathscr{T}}\sum _{i=1,\mathrm{...},N}({\hat{a}}_{{\overrightarrow{R}}_{i}}^{\dagger }{\hat{a}}_{{\overrightarrow{R}}_{i}}+{\hat{b}}_{{\overrightarrow{R}}_{i}+{\overrightarrow{\tau }}_{1}}^{\dagger }{\hat{b}}_{{\overrightarrow{R}}_{i}+{\overrightarrow{\tau }}_{1}})-{\mathscr{T}}\,^{\prime} \sum _{\begin{array}{c}i=1,\mathrm{...},N\\ l=1,2,3\end{array}}({e}^{i\frac{e}{\hslash }\overrightarrow{A}\cdot {\overrightarrow{\tau }}_{l}}\,{\hat{a}}_{{\overrightarrow{R}}_{i}}^{\dagger }{\hat{b}}_{{\overrightarrow{R}}_{i}+{\overrightarrow{\tau }}_{l}}+{e}^{-i\frac{e}{\hslash }\overrightarrow{A}\cdot {\overrightarrow{\tau }}_{l}}\,{\hat{b}}_{{\overrightarrow{R}}_{i}+{\overrightarrow{\tau }}_{l}}^{\dagger }{\hat{a}}_{{\overrightarrow{R}}_{i}}).$$

Here ${\mathscr{T}}$ and ${\mathscr{T}}\,^{\prime}$ are the diagonal and nearest-neighbor off-diagonal matrix elements of the Hamiltonian respectively in the basis of atomic orbitals in absence of $\overrightarrow{A}$; $({\hat{a}}_{{\overrightarrow{R}}_{i}},{\hat{a}}_{{\overrightarrow{R}}_{i}}^{\dagger })$ and $({\hat{b}}_{{\overrightarrow{R}}_{i}+{\overrightarrow{\tau }}_{l}},{\hat{b}}_{{\overrightarrow{R}}_{i}+{\overrightarrow{\tau }}_{l}}^{\dagger })$ are annihilation and creation operators for the two sublattices in graphene, with ${\overrightarrow{R}}_{i}$, *i* = 1, …, *N* denoting the sublattice sites, and *τ*_*l*_, *l* = 1, 2, 3 denoting the vectors from a lattice site to its three nearest neighbors. The current density operator can then be obtained by $\hat{\overrightarrow{j}}({\overrightarrow{R}}_{i})=\partial \hat{H}/\partial \overrightarrow{A}$^16^.

In weak electromagnetic (EM) fields, both $\hat{H}$ and $\hat{\overrightarrow{j}}$ can be expanded in terms of $\overrightarrow{A}$. $\hat{H}$ can be broken into a non-interacting part ${\hat{H}}_{0}$ and an interacting part ${\hat{H}}_{I}$, with ${\hat{H}}_{I}={\hat{H}}_{I}^{(1)}+{\hat{H}}_{I}^{(2)}+\cdots$ and the superscripts indicating the order of $\overrightarrow{A}$:

2 $${\hat{H}}_{0}={\mathscr{T}}\sum _{i=1,\mathrm{...},N}({\hat{a}}_{{\overrightarrow{R}}_{i}}^{\dagger }{\hat{a}}_{{\overrightarrow{R}}_{i}}+{\hat{b}}_{{\overrightarrow{R}}_{i}+{\overrightarrow{\tau }}_{1}}^{\dagger }{\hat{b}}_{{\overrightarrow{R}}_{i}+{\overrightarrow{\tau }}_{1}})-{\mathscr{T}}^{\prime} \sum _{\begin{array}{c}i=1,\mathrm{...},N\\ l=1,2,3\end{array}}({\hat{a}}_{{\overrightarrow{R}}_{i}}^{\dagger }{\hat{b}}_{{\overrightarrow{R}}_{i}+{\overrightarrow{\tau }}_{l}}+{\hat{b}}_{{\overrightarrow{R}}_{i}+{\overrightarrow{\tau }}_{1}}^{\dagger }{\hat{a}}_{{\overrightarrow{R}}_{i}}),$$

3 $${\hat{H}}_{I}^{(1)}=-\,i\frac{e{\mathscr{T}}\,^{\prime} }{\hslash }\sum _{\begin{array}{c}i=1,\mathrm{...},N\\ l=1,2,3\end{array}}\,\overrightarrow{A}({\overrightarrow{R}}_{i},t)\cdot {\overrightarrow{\tau }}_{l}({\hat{a}}_{{\overrightarrow{R}}_{i}}^{\dagger }{\hat{b}}_{{\overrightarrow{R}}_{i}+{\overrightarrow{\tau }}_{l}}-{\hat{b}}_{{\overrightarrow{R}}_{i}+{\overrightarrow{\tau }}_{l}}^{\dagger }{\hat{a}}_{{\overrightarrow{R}}_{i}}),$$

4 $${\hat{H}}_{I}^{(2)}=\frac{{e}^{2}{\mathscr{T}}^{\prime} }{2{\hslash }^{2}}\sum _{\begin{array}{c}i=1,\mathrm{...},N\\ l=1,2,3\end{array}}\,{[\overrightarrow{A}({\overrightarrow{R}}_{i},t)\cdot {\overrightarrow{\tau }}_{l}]}^{2}({\hat{a}}_{{\overrightarrow{R}}_{i}}^{\dagger }{\hat{b}}_{{\overrightarrow{R}}_{i}+{\overrightarrow{\tau }}_{l}}+{\hat{b}}_{{\overrightarrow{R}}_{i}+{\overrightarrow{\tau }}_{l}}^{\dagger }{\hat{a}}_{{\overrightarrow{R}}_{i}}).$$

and similarly $$\hat{\overrightarrow{j}}={\hat{\overrightarrow{j}}}_{0}+{\hat{\overrightarrow{j}}}^{(1)}+{\hat{\overrightarrow{j}}}^{(2)}+\cdot \cdot \cdot $$:

5 $${\hat{\overrightarrow{j}}}_{0}({\overrightarrow{R}}_{i})=i\frac{e{\mathscr{T}}\,^{\prime} }{\hslash }\sum _{l=1,2,3}\,{\overrightarrow{\tau }}_{l}({\hat{a}}_{{\overrightarrow{R}}_{i}}^{\dagger }{\hat{b}}_{{\overrightarrow{R}}_{i}+{\overrightarrow{\tau }}_{l}}-{\hat{b}}_{{\overrightarrow{R}}_{i}+{\overrightarrow{\tau }}_{l}}^{\dagger }{\hat{a}}_{{\overrightarrow{R}}_{i}}),$$

6 $${\hat{\overrightarrow{j}}}^{(1)}({\overrightarrow{R}}_{i})=-\,\frac{{e}^{2}{\mathscr{T}}\,^{\prime} }{{\hslash }^{2}}\sum _{l=1,2,3}\,[\overrightarrow{A}({\overrightarrow{R}}_{i},t)\cdot {\overrightarrow{\tau }}_{l}]{\overrightarrow{\tau }}_{l}({\hat{a}}_{{\overrightarrow{R}}_{i}}^{\dagger }{\hat{b}}_{{\overrightarrow{R}}_{i}+{\overrightarrow{\tau }}_{l}}+{\hat{b}}_{{\overrightarrow{R}}_{i}+{\overrightarrow{\tau }}_{l}}^{\dagger }{\hat{a}}_{{\overrightarrow{R}}_{i}}),$$

7 $${\hat{\overrightarrow{j}}}^{(2)}({\overrightarrow{R}}_{i})=-\,i\frac{{e}^{3}{\mathscr{T}}\,^{\prime} }{2{\hslash }^{3}}\sum _{l=1,2,3}\,{[\overrightarrow{A}({\overrightarrow{R}}_{i},t)\cdot {\overrightarrow{\tau }}_{l}]}^{2}{\overrightarrow{\tau }}_{l}({\hat{a}}_{{\overrightarrow{R}}_{i}}^{\dagger }{\hat{b}}_{{\overrightarrow{R}}_{i}+{\overrightarrow{\tau }}_{l}}-{\hat{b}}_{{\overrightarrow{R}}_{i}+{\overrightarrow{\tau }}_{l}}^{\dagger }{\hat{a}}_{{\overrightarrow{R}}_{i}}).$$

Crucially, the Peierls substitution yields terms in Eqs (4), (6) and (7), which cannot be obtained by replacing $\hat{\overrightarrow{p}}$ with $\hat{\overrightarrow{p}}+e\overrightarrow{A}$ in the Dirac Hamiltonian (as done in previous works^8,9,11^). In fact, it is the term in Eq. (6) that cancels out a term in Eq. (5) that could otherwise cause a divergence in the linear conductivity^11^ (see details in the Methods).

**Figure 1:**
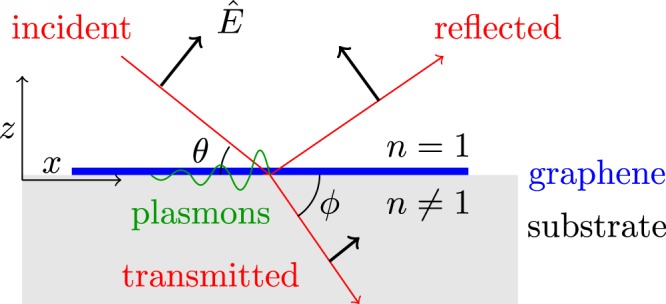
Illustration of electromagnetic fields ($\overrightarrow{E}$) (applicable to pump, probe, and DFG) propagating in the x-z plane. All the fields are p-polarized and the directions of propagation and polarizations are indicated by the red and black arrows respectively. The angles of incidence and transmission are defined in the figure as *θ* and *ϕ*.

At the Dirac points, following standard procedures, one can derive equivalent spinor forms of the above operators. The expectation value of the current density in the presence of the fields can be calculated as $\langle \hat{\overrightarrow{j}}\rangle ={\rm{Tr}}(\hat{\rho }\,\hat{\overrightarrow{j}})$, where $\hat{\rho }$ is the (self-consistent) single-particle density matrix. The matrix elements can be calculated by using the time evolution equation ${\rm{d}}\hat{\rho }/dt=(i/\hslash )[\hat{\rho },{\hat{H}}_{0}+{\hat{H}}_{I}]$ and solving the density matrix $\hat{\rho }={\sum }_{n}\,{\hat{\rho }}^{(n)}$ perturbatively in powers of ${\hat{H}}_{I}$. We leave the detailed derivations of the spinor formalism and the currents to the Methods, and only quote the result for the nonlinear current here. We consider the response to EM fields described by a potential $\overrightarrow{A}(\overrightarrow{r},t)=(1/2){\sum }_{m=1,2}\,[{A}_{m}\hat{x}\,{e}^{i({q}_{m}x-{\omega }_{m}t)}+{\rm{c}}.{\rm{c}}.]$, where the electric field components parallel to the graphene layer are related to the potentials by ${E}_{mx}=-\,\partial {A}_{m}/\partial t$; *q*_*m*_ and *ω*_*m*_ are the wavevectors and angular frequencies, and $\hat{x}$ is a unit vector along the x-direction. Following relevant experiment^14^ the fields for *m* = 1, 2 are called “pump” and “probe” respectively, and we illustrate their configurations in Fig. (1). Here we are interested in the case of DFG and look for the nonlinear current at difference frequency *ω*_3_ = *ω*_2_ − *ω*_1_ and wavevector *q*_3_ = *q*_2_ − *q*_1_, which can be formally written as ${j}_{x}^{(2)}={\sigma }^{(2)}{E}_{1x}{E}_{2x}$, where *σ*^(2)^ defines the second-order conductivity. Under the relevant experimental conditions^7,14^ *ω*_1_ ≈ *ω*_2_ ≫ *ω*_3_,

8 $${\sigma }^{(2)}({\omega }_{3},{q}_{3})\approx -\,\frac{2{e}^{3}{v}_{F}^{2}}{\pi {\hslash }^{2}}(\frac{{q}_{3}}{{\omega }_{3}})\frac{{\omega }_{F}^{4}}{({\omega }_{3}^{2}-4{\omega }_{F}^{2}){\omega }_{2}^{4}},$$

where in *ω*_*F*_ and *v*_*F*_ are the Fermi angular frequency and velocity.

### Comparison of perturbation theory with experiment

**Figure 2:**
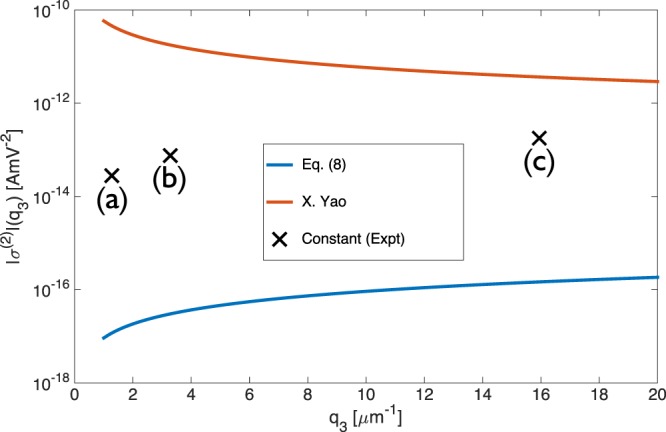
Comparison of the nonlinear conductivity ${\sigma }^{(2)}({\omega }_{3},{q}_{3})$ derived here from Eq. (8) with other results derived using perturbation theory: Eq. (5) from X. Yao^6^. All theoretical curves are plotted for a Fermi energy of 500 meV and difference frequency of 15.3 THz. [Experimental estimates from Constant^14^, for the different experimental geometries (a), (b), (c) (Table 1) are indicated by black crosses].

X. Yao^6^ were the first to derive the nonlinear conductivity in graphene relevant to DFG. In Fig. 2, we plot this derived second order nonlinear response (Eq. (5) of X. Yao^6^ converted to *σ*^(2)^) along with experimental results from Constant^14^, recent perturbative calculations^11^ and our own Eq. (8). It is important to note first that the results of Wang^11^ and Cheng^9^ strongly agree with our own suggesting this is the correct prediction from pertubation theory (in addition Tokman^8^ and Rostami^17^ achieve the same result except a factor of 2 which may be due to definitions we could not clearly identify). However there is a rather large discrepancy between Eq. (8) and the model derived by X. Yao^6^, which were derived for identical conditions using perturbation theory. Moreover the conductivity derived by X. Yao^6^ has a non-physical divergence for ${q}_{3}\to 0$. While it is not clear from where this unphysical behavior arises, in a centrosymmetric material such as graphene this behavior is paradoxical. Meanwhile, the conductivity from Eq. (8) tends to zero as ${q}_{3}\to 0$, as it must in graphene. We note that, depending on the value of *q*_3_ in Fig. 2, the magnitude of *σ*^(2)^ predicted by Eq. (8) is at least 4 orders lower than that found by X. Yao^6^.

The first experimental signatures attributed to DFG of plasmons were found by Constant^14^. In this experiment, by illuminating the graphene with two tunable, femtosecond laser pulses (“pump” and “probe”) with well-defined angles of incidence but different frequencies, Constant^14^ was able to phase-match to the plasmon. The geometry of the experiment is the same as that chosen for our theoretical calculation defined in Fig. 1. The graphene supports tightly guided plasmons with a dispersion relation *ω*_*pl*_(*k*). The differential reflectance of the probe pulse Δ*R* was seen to change significantly whenever the difference frequency and wavevector were aligned to the plasmon dispersion relation, ${\omega }_{pl}({\overrightarrow{k}}_{1}-{\overrightarrow{k}}_{2})={\omega }_{1}-{\omega }_{2}$, suggesting efficient plasmon excitation via DFG. In practice, a range of difference frequencies and wavevectors were scanned by continuously varying the pump wavelength, and by choosing different discrete incident beam angles.

Constant investigated three experimental geometries (noted in Table 1) with different angles of incidence, *θ*. We examine one of the resonant conditions for each of the three experimental geometries, as defined in Table 1. Assuming the differential reflection signals arise from DFG, one can use the model introduced in the Supplementary Information of Constant^14^ (briefly reviewed in the Methods section) to estimate a value for *σ*^(2)^ for each measurement. The results of this analysis, i.e. values of *σ*^(2)^ which describe the experimental signals, are also shown in Table 1.

**Table 1:** Differential reflectivity, normalized to pump fluence Φ, and experimentally determined *σ*^(2)^ extracted using the model of^14^ for three geometries (a), (b) and (c). For all geometries *λ*_*probe*_ = 617.53 nm.

Geometry	*θ*_*pump*_	*θ*_*probe*_	*λ*_*pump*_ (nm)	$\frac{{\omega }_{3}}{2\pi }$ (THz)	$\frac{{\rm{\Delta }}R}{R{\rm{\Phi }}}$ (mJ^−1^ cm^2^)	*σ*^(2)^ (fAmV^−2^)
(a)	45°	55°	607	7.0	−0.0097	24
(b)	50°	70°	597	15.3	−0.025	75
(c)	125°	15°	587	23.8	0.062	180

Figure 2 compares the experimental values of *σ*^(2)^ from Constant^14^ and theoretical predictions from Eq. (8), Wang^11^ and X. Yao^6^. Firstly, the *q*_3_ dependence of Yao^6^ clearly differs greatly from that of both the experiment and the near-linear predictions of other theoretical derivations. The experimental magnitudes of *σ*^(2)^ are also significantly lower than the prediction of X. Yao^6^, and several orders higher than those from recent perturbative works^8,9,11^. As found by^17^, it is only possible to find agreement between the experiments^14^ and perturbative second order calculations if one invokes an unphysically low decay rate for the plasmon (resulting in extraordinarily narrow resonances). More recently, a similar experiment has been carried out by B. Yao^7^ in a waveguiding geometry, and the theory from X. Yao^6^ was used to model the experimental signals. While the geometries of B. Yao^7^ and Constant^14^ are significantly different, similar signals were observed in each experiment. Therefore, ignoring the unphysical results in X. Yao^6^, the large discrepancy between both experiments and the theoretical consensus points to a second order response that is not purely perturbative, as originally interpreted. In the remainder of this paper, we therefore discuss other possible contributions which might account for the discrepancy between perturbation theory and the experiments of B. Yao^7^ and Constant^14^.

### Substrate Response

In this section we consider contribution of the second order nonlinearity of the quartz substrate used in experiment^14^. The analysis significantly simplifies if the nonlinear polarization is generated far from a phase-matching condition of the bulk, and depletion can be ignored, as should be the situation for Constant^14^. In this case, the pump and probe fields generate a polarization in quartz are given by

9 $${P}_{3}(\overrightarrow{r},t)={\varepsilon }_{0}{\chi }^{(2)}{e}^{i[({\overrightarrow{k}}_{T1}-{\overrightarrow{k}}_{T2})\cdot \overrightarrow{r}-({\omega }_{1}-{\omega }_{2})t]}{t}_{1}{t}_{2}^{\ast }{E}_{I1}{E}_{I2}^{\ast }\,,$$

where *χ*^(2)^ is the second order susceptibility of the substrate, ${\overrightarrow{k}}_{Ti}$, *t*_*i*_, and *E*_*Ii*_ denote the wavevector on the transmitted (substrate) side, transmission coefficient, and the incident field amplitude of the pump (*i* = 1) and the probe (*i* = 2) fields respectively. The transmission coefficients *t*_*i*_ are given in the Methods. The subscript *i* = 3 indicates quantities corresponding to the difference frequency signal at *ω*_3_ = *ω*_1_ − *ω*_2_. As charge density waves in graphene are driven by an electric field, we must relate the nonlinear polarization to the field generated in the quartz, which satisfies the wave equation

10 $$-\,{\nabla }^{2}{E}_{3s}+\frac{{\varepsilon }_{3}}{{c}^{2}}\frac{{\partial }^{2}{E}_{3s}}{\partial {t}^{2}}=-\,{\mu }_{0}\frac{{\partial }^{2}{P}_{3}}{\partial {t}^{2}}.$$

Here, the subscript “s” denotes that this is an effective source field that will later drive a response in the graphene (distinct from the resulting plasmon field). Also, ${\varepsilon }_{3}=n{({\omega }_{3})}^{2}$ indicates the permittivity of quartz evaluated at the difference frequency, with the model of the frequency dependent *n*(*ω*) given in the Appendix. Due to the plane-wave nature of *P*_3_, *E*_3*s*_ takes on the same spatial and frequency dependence. In our regime of interest, the spatial derivative of the field, $|{\nabla }^{2}{E}_{3s}|=|{\overrightarrow{k}}_{T1}-{\overrightarrow{k}}_{T2}{|}^{2}{E}_{3s}$, is significantly larger than the time derivative. This is because the pump and probe fields are chosen to phase-match with surface plasmons in graphene (thus the associated wavevectors are much larger than free-space fields at the difference frequency). Thus the field amplitude created by the nonlinear polarization is well approximated by

11 $${E}_{3s}\approx \frac{{({\omega }_{1}-{\omega }_{2})}^{2}}{{c}^{2}|{\overrightarrow{k}}_{T1}-{\overrightarrow{k}}_{T2}{|}^{2}}{\chi }^{(2)}{t}_{1}{t}_{2}^{\ast }{E}_{I1}{E}_{I\,2}^{\ast }.$$

In particular, it should be noted that a large wavevector mismatch results in strong suppression of the field. To simplify the discussion, we will assume the scenario which produces the highest field, i.e. in which *E*_3*s*_ is completely polarized along $\hat{x}$ (parallel to the graphene sheet) so that it maximally drives a charge density wave in graphene. As we see below, even in this best case scenario, the generated field is rather small.

Since the nonlinear response is considered here to be completely within the substrate, which provides an effective source field *E*_3*s*_, the remaining part of the calculation is completely linear in its nature. Using the conventions in Fig. 1, we take “reflected” and “transmitted” field components of unknown amplitude, which correspond to the plasmon field on the vacuum and substrate sides. The wavevector along $\hat{x}$ for these fields is equal to *q*_3_ = *q*_*T*1_ − *q*_*T*2_, where *q*_*T*1_ and *q*_*T*2_ are the in-plane components of ${\overrightarrow{k}}_{T1}$ and ${\overrightarrow{k}}_{T2}$, while the perpendicular components must satisfy the respective dispersion relations for each side of the interface, *e.g*., ${k}_{T3z}^{2}={\varepsilon }_{3}{({\omega }_{3}/c)}^{2}-{q}_{3}^{2}$. Similar to the procedures to solve the pump (probe) field laid out in the methods, the two unknown field amplitudes can be readily solved by taking *E*_3*s*_ to be the incident field on the substrate side, and enforcing electromagnetic boundary conditions at the vacuum-graphene-quartz interface, which yields the following parallel-field component on the substrate side, evaluated at the graphene layer (z = 0),

12 $${E}_{pl}=-\,{E}_{3s}\frac{(c{\varepsilon }_{0}+{\sigma }^{(1)}({\omega }_{3})\,\sin \,{\theta }_{3})\,\sin \,{\varphi }_{3}}{c{\varepsilon }_{0}\,\sin \,{\varphi }_{3}+\,\sin \,{\theta }_{3}(c{\varepsilon }_{0}{n}_{3}+{\sigma }^{(1)}({\omega }_{3})\,\sin \,{\varphi }_{3})}.$$

Here, *σ*^(1)^(*ω*_3_) is the linear conductivity of graphene evaluated at frequency *ω*_3_.

Specifically we can numerically evaluate *E*_*pl*_ for geometry (b) in^14^. Taking a value of ${\chi }^{\mathrm{(2)}}=0.3\,{{\rm{pmV}}}^{-1}$ for quartz^18^, we find that ${E}_{pl}\approx 15\,{{\rm{Vm}}}^{-1}$. The modeling in^14^ predicts a considerably larger value for the inferred plasmon field in experiment of $\approx 8\times {10}^{4}\,{{\rm{Vm}}}^{-{\rm{1}}}$. We therefore do not believe that the substrate nonlinearity contributes significantly to the signals observed in^14^. However, we note that our substrate model does not consider any surface enhanced nonlinearity. Its theoretical modeling would require experimental measurements of surface nonlinear coefficients relevant to our system, which we were unable to find in literature.

### Photothermal Effect

Here, we present an alternative mechanism by which a plasmon field at the difference frequency and wavevector can be generated. Fundamentally, the effect discussed below arises from the linear Seebeck effect, so that the total current is described by

13 $${j}_{x}({\omega }_{3},{q}_{3})={\sigma }^{\mathrm{(1)}}({\omega }_{3},{q}_{3}){E}_{3x}+{\sigma }^{\mathrm{(2)}}{E}_{1x}{E}_{2x}+{\sigma }^{\mathrm{(1)}}S\frac{dT}{dx}.$$

The first term on the right hand side describes the normal linear relationship between the current and field, while the second describes the conventional second order electronic nonlinearity. The third term, and most important here, arises due to photothermal effects, and accounts for the Seebeck current emerging due to a temperature gradient $(\frac{dT}{dx})$ in a material described by Seebeck coefficient S. As we discuss below, this term can give rise difference frequency currents even in the absence of a nonlinear conductivity (i.e. even when *σ*^(2)^ = 0).

**Figure 3:**
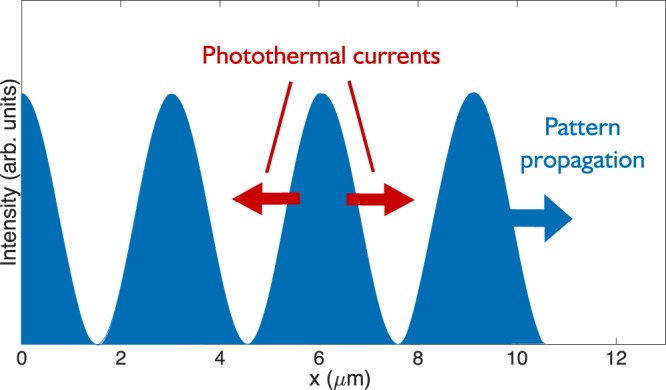
Intensity pattern generated from the interference of two beams in geometry (b) (*θ*_*pump*_ = 70°, *θ*_*probe*_ = 50° *λ*_*pump*_ = 587 nm, *λ*_*probe*_ = 617.53 nm). The temperature gradient in the sample follows this pattern and generates photothermal currents when thermalising. The pattern propagates, phase-matched to the difference frequency field, with wavevector *q*_3_ = *q*_1_ − *q*_2_.

It is known that excitation of graphene carriers by intense femtosecond pulses (${\rm{\Phi }}\approx 0.1\,{{\rm{mJcm}}}^{-2}$ with pulse width ~100 fs in^14^) is not perturbative in nature. The electron temperature is raised by several thousand kelvin under such excitation^19^ and is not in equilibrium with the phonon temperature. Furthermore, under geometries similar to those used in B. Yao^7^ and Constant^14^, heating due to optical illumination is not homogeneous. When two or more light sources of similar frequency are incident on an interface at oblique angles, the result will be a near stationary interference pattern such as that shown in Fig. 3. When the frequencies are slightly different, the pattern will propagate in the plane with a velocity equal to *ω*_3_/*q*_3_. The spatially dependent interference pattern will give rise to a temperature modulation that propagates along the graphene sheet, which, via the Seebeck effect, can also generate a current and thereby drive a plasmon excitation when phase-matching conditions are satisfied. Below, we present a theoretical model of this effect, and show that it can generate a difference frequency field that is orders of magnitude larger than that calculated from the perturbation theory.

The intensity pattern imprinted on the graphene sheet can be expressed as

14 $$\begin{array}{c}I(x,t)=\frac{c{\varepsilon }_{0}}{2}{|{E}_{1x}{e}^{i({q}_{1}x-{\omega }_{1}t)}+{E}_{2x}{e}^{i({q}_{2}x-{\omega }_{2}t)}|}^{2}\\ \,\,\,\,\,\approx \frac{c{\varepsilon }_{0}{{E}_{1x}}^{2}}{2}[1+2|\frac{{E}_{2x}}{{E}_{1x}}|\cos ({q}_{3}x-{\omega }_{3}t)]+{\mathscr{O}}({E}_{2x}^{2})\mathrm{.}\end{array}$$

Here *E*_1*x*_ and *E*_2*x*_ are the in-plane components of the pump and probe fields respectively, and we have assumed the probe field is much weaker than the pump field. The ratio of the in-plane components can be calculated as $|{E}_{2x}/{E}_{1x}|=({t}_{2}^{(L)}\,\sin \,{\varphi }_{2})/({t}_{1}^{(L)}\,\sin \,{\varphi }_{1})\sqrt{{I}_{2}/{I}_{1}}$, with *I*_1_ and *I*_2_ being the incident intensities of the pump and probe beams respectively, and the linear transmission coefficient ${t}_{i}^{(L)}(i=1,2)$ is given by Eq. (38) considering only the linear optical conductivity of graphene Eq. (40). Using the parameters in the experiment of Constant^14^, we obtain $|{E}_{2x}/{E}_{1x}|\approx 0.1$.

The intensity pattern acts as a heat source for the temperature distribution which, in linear response theory, satisfies a diffusion equation:

15 $$\frac{\partial T}{\partial t}-\alpha \frac{{\partial }^{2}T}{\partial {x}^{2}}=\beta I(x,t)-y(T-{T}_{0}),$$

where *α* is the diffusivity, *β* is the heating rate due to the intensity pattern, and *y* is the relaxation rate back to the equilibrium temperature *T*_0_. Due to the linearity of the equation, it can be readily solved in the Fourier domain, in which when taking into account Eq. (14) the solution takes the form

16 $$T(x,t)={T}_{0}+{T}_{dc}+{T}_{ac}\,\cos ({q}_{3}x-{\omega }_{3}t-{\rm{\Psi }}).$$

Here, *T*_*dc*_ is the (large) position- and time-independent temperature increase arising from the incident lasers, while *T*_*ac*_ represents a position- and time-varying temperature oscillation that must necessarily be generated in the presence of moving intensity interference pattern. Ψ denotes a phase offset between the intensity and temperature modulations, whose specific form is not relevant here. Substituting *T*(*x*, *t*) into Eq. (15), in the regime of interest *y* ≪ *ω*_3_, one obtains |*T*_*ac*_|/*T*_*dc*_ ≈ 0.2*y*/*ω*_3_. As expected, the temperature modulation *T*_*ac*_ is reduced significantly as the oscillation frequency *ω*_3_ increases with respect to the damping rate. It is known that intense, femtosecond pulses similar to those in^14^ lead to *T*_*dc*_ of approximately 2000 K^19^. The relaxation rate *y* is due to electron-phonon scattering, and we take a value of *y* ≈ 1/(100 fs)^19^. At *ω*_3_ = 2*π* × 10 THz these parameters give a temperature modulation *T*_*ac*_ = 60 K.

The Seebeck effect enables the generation of a source current in the presence of a temperature gradient, and this can be described by ${j}_{s}({\omega }_{3},{q}_{3})={\sigma }^{(1)}({\omega }_{3}){q}_{3}S|{T}_{ac}|$, where *S* is called the Seebeck coefficient. In principle, the Seebeck coefficient could be frequency and wavevector dependent. However, this dependence has not been measured carefully in literature, nor is it straightforward to calculate from first principles. There have been several measurements of the Seebeck effect in graphene, both in DC experiments (*S* ≈ 5 × 10^−5^ V/K^20^, *S* ≈ 8 × 10^−5^ V/K^21^) and under illumination from 100 fs pulses (S ≈ 10^−4^ V/K^22^). Whilst it is hard to predict how the Seebeck effect behaves on 10 fs timescales relevant here (corresponding to peak to peak propagation time of the intensity pattern in Fig. 3), it is likely that photothermal effects will be higher on ballistic timescales, as with other materials^23^. Here we use a conservative value of *S* ≈ 10^−4^ V/K reported in^22^.

Now using the standard EM boundary conditions at the graphene layer (see Fig. 1), with the aid of the charge continuity equation, one can find the relation between the electric fields and the surface current density at the difference frequency *ω*_3_. Note now the Seebeck effect contribution needs to be added to the surface current density:

17 $${j}_{x}({\omega }_{3},{q}_{3})={\sigma }^{(1)}({\omega }_{3}){E}_{3x}+{j}_{s}({\omega }_{3},{q}_{3}).$$

Then solving the equations of the boundary conditions (see Methods for more details), we obtain for the electric field at the difference frequency:

18 $${E}_{3x}=-\,\frac{{t}_{3}^{(L)}}{2c{\varepsilon }_{0}}{\sigma }^{(1)}({\omega }_{3}){q}_{3}S|{T}_{ac}|\sin \,{\varphi }_{3},$$

where ${t}_{3}^{(L)}$ is the linear transmission coefficient at frequency and wavevector *ω*_3_, *q*_3_. For geometry (b) of Constant^14^ we find a magnitude of *E*_3*x*_, when on plasmon resonances, of ${E}_{pl}\approx 2.3\times {10}^{3}$ V/m.

Just as the pump and probe fields can generate a plasmon field through the Seebeck effect, a back-action effect (involving Seebeck mixing of the plasmon and pump fields) results in a change of the probe differential reflectance. In principle, this could be rigorously calculated in a manner similar to above, but this would require knowledge of the Seebeck coefficient at *optical* frequencies, which has never been measured or calculated. However, we can nonetheless obtain an approximate value for the differential reflection, by exploiting conservation of energy. In particular, in steady state, the number of plasmons dissipated per unit time must equal the rate of photons removed from (added to) the pump (probe) beam. There are two contributions to the energy dissipation at the difference frequency: both the graphene layer and the substrate will exhibit absorption. For graphene, the power loss per unit area from absorption can be found by ${P}_{g}=(1/2)\,\mathrm{Re}\,{\sigma }^{(1)}({\omega }_{3}){|{E}_{3x}|}^{2}$^[ 24^. In the experiment, the substrate itself (quartz) can provide a non-negligible loss, through coupling with phonons. The corresponding power loss per unit area can be calculated as ${P}_{s}=({\varepsilon }_{0}/2){\int }_{-\infty }^{0}\,dz\,{\omega }_{3}\,{\rm{Im}}\,[{n}^{2}({\omega }_{3})]\,{|{E}_{3}|}^{2}$, where our model for the frequency-dependent refractive index *n*(*ω*) is provided in the Methods. Then, the number of photons, at the difference frequency, absorbed per unit time and area is ${{\rm{\Gamma }}}_{d}=({P}_{g}+{P}_{s})/\hslash {\omega }_{3}$. At the individual photon level of a DFG process, an incoming pump photon breaks down to an outgoing probe photon and a plasmon, and therefore the number of plasmons created is also equal to the number of newly generated probe photons that enter either the reflected or transmitted beam. The number of photons per unit time and area in the incident probe beam is simply ${{\rm{\Gamma }}}_{in}={I}_{2}\,\sin \,{\theta }_{2}/\hslash {\omega }_{2}$. Thus the order of magnitude of the differential reflectance of the probe beam can be estimated as ${\rm{\Delta }}R/Rn\sim {{\rm{\Gamma }}}_{d}{/{\rm{\Gamma }}}_{in}$. For configuration (b) in Table 1, we estimate the peak differential reflectance after normalized by the fluence (0.1 mJ cm^−2^) to be ~7 × 10^−7^ mJ^−1^ cm^2^. Although we emphasize that the Seebeck effect and the electronic nonlinearity *σ*^(2)^ are completely independent effects, nonetheless to facilitate a better comparison, one can ask what hypothetical value of nonlinear conductivity ${\sigma }_{S}^{(2)}$ would be required, in order to produce the same current as predicted from the Seebeck effect, i.e. ${j}_{s}={\sigma }_{S}^{(2)}{E}_{1x}{E}_{2x}^{\ast }$. We extract a value of ${\sigma }_{S}^{\mathrm{(2)}}\approx 3.1\times {10}^{-15}\,{{\rm{AmV}}}^{-2}$.

While this model predicts a value slightly smaller than experiment (${\sigma }^{\mathrm{(2)}}\approx 7.5\times {10}^{-14}{{\rm{AmV}}}^{-2}$^[ 14^), the Seebeck coefficient could be larger on ballistic timescales relevant here (≈10 fs peak to peak propagation time) as is expected for other materials^23^. Nevertheless such an effect is fundamental to the experiments and is significantly larger than the predictions of perturbation theory.

We note that photothermal effects will be prominent in the waveguiding geometry of B. Yao^7^ (in such a geometry, even though the absolute field intensities are lower, the considerably larger propagation length can compensate). Interestingly, the power dependence of such a photothermal signal would not necessarily follow that of conventional difference frequency generation ($j={\sigma }^{(2)}{E}_{1}{E}_{2}^{\ast }$), and could explain those observed by Constant^25^. Investigating the intensity dependences of these nonlinear signals could provide great insight into the origins of these effects. We also note that the photo-Dember effect can similarly induce local intensity dependent currents and is surprisingly large in graphene on ultrafast timescales^26^. However, since the photo-Dember effect depends on mobility asymmetry between electrons and holes, it will be sample and substrate specific, making it difficult to estimate.

## Conclusions

We have derived a second order conductivity of planar graphene (${\sigma }^{\mathrm{(2)}}\approx {10}^{-17}\,\,{{\rm{AmV}}}^{-{\rm{2}}}$) with non-local perturbation theory, addressing the long wavelength divergence in^6,7^ and divergent linear current in^11^. However, while our result is in agreement with recent calculations^8,9,11,17^, it is insufficient to explain observations from experiment^7,14^. We also show that this discrepancy cannot be attributed to the bulk nonlinearity of the substrate.

We also discuss the possibility of photothermal effects in experiments, wherein a spatial intensity pattern resulting from interference of incident beams leads to local inhomogeneous heating of the sample and show that these effects will give rise to frequency mixing currents. We derive a rigorous model for DFG arising from photothermal effects (with the only uncertainties arising from knowledge of material properties such as the Seebeck coefficient), and conservatively estimate a DFG current which is at least two orders of magnitude larger than that found from perturbation theory, significantly closer to experimental estimates from^14^. Microscopic modeling of such local photothermal effects (and other non-equilibrium processes) presents a considerable challenge, and it would be interesting to develop theoretical techniques to do so. We believe that such efforts would shed further light on discrepancies between recent experiments^7,14^ and theory^6–11^ for all-optical plasmon generation processes in graphene, and enable the strengths of these nonlinear processes to be optimized for future nonlinear optical applications.

## Methods

### Spinor formalism

We can perform a Fourier expansion on the operators $({\hat{a}}_{{\overrightarrow{R}}_{i}},{\hat{a}}_{{\overrightarrow{R}}_{i}}^{\dagger })$ and $({\hat{b}}_{{\overrightarrow{R}}_{i}+{\overrightarrow{\tau }}_{l}},{\hat{b}}_{{\overrightarrow{R}}_{i}+{\overrightarrow{\tau }}_{l}}^{\dagger })$ in the tight-binding Hamiltonian in terms of the operators in the reciprocal lattice space:

19 $${\hat{a}}_{{\overrightarrow{R}}_{i}}=\frac{1}{\sqrt{N}}\sum _{\overrightarrow{k}\in {{\rm{\Omega }}}_{B}}\,{\hat{a}}_{\overrightarrow{k}}\,{e}^{i\overrightarrow{k}\cdot {\overrightarrow{R}}_{i}},\,{\rm{and}}\,{\hat{b}}_{{\overrightarrow{R}}_{i}+{\overrightarrow{\tau }}_{l}}=\frac{1}{\sqrt{N}}\sum _{\overrightarrow{k}\in {{\rm{\Omega }}}_{B}}\,{\hat{b}}_{\overrightarrow{k}}\,{e}^{{\rm{i}}\overrightarrow{k}\cdot ({\overrightarrow{R}}_{i}+{\overrightarrow{\tau }}_{l})},$$

in which Ω_*B*_ denotes the first Brillouin zone, $\overrightarrow{k}$ is the electron momentum and *N* is the number of sites in one sublattice. One can then substitute these expansions into the expressions of the Hamiltonian Eqs (2–4) and current density operators Eqs (5–7). As usual, near the two Dirac points $\overrightarrow{K}$ and $-\,\overrightarrow{K}$^27^, the operators can be expanded in orders of $\overrightarrow{k}$ referenced from $\overrightarrow{K}$ or $-\,\overrightarrow{K}$: $\overrightarrow{k}\mp \overrightarrow{K}\to \overrightarrow{k}$. One can then derive equivalent spinor forms for Eqs (2–7) in the first quantization picture. If only the terms to lowest order of $\overrightarrow{k}$ are kept, then

20 $${\hat{H}}_{0}\to {v}_{F}\hat{\overrightarrow{\sigma }}\cdot \hat{\overrightarrow{p}}\,{\rm{at}}\,\overrightarrow{K},\,{\rm{and}}\,-\,{v}_{F}{\hat{\overrightarrow{\sigma }}}^{\ast }\cdot \hat{\overrightarrow{p}}\,{\rm{at}}-\,\overrightarrow{K},$$

in which the Fermi velocity ${v}_{F}=\sqrt{3}a{\mathscr{T}}\,^{\prime} /2\hslash$, with *a* being the lattice constant of the underlying sublattices, and $\hat{\overrightarrow{\sigma }}\equiv {\hat{\sigma }}_{x}\hat{x}+{\hat{\sigma }}_{y}\hat{y}$ with ${\hat{\sigma }}_{x,y}$ representing the Pauli spin matrices. Meanwhile, for ${\hat{H}}_{I}$:

21 $${\hat{H}}_{I}^{(1)}\to \pm e{v}_{F}A(\overrightarrow{r},t){\hat{\sigma }}_{x},$$

with “+” at $\overrightarrow{K}$ and “−” at $-\,\overrightarrow{K}$, and we have for simplicity assumed $\overrightarrow{A}$ is along the *x*-axis. The second order component is meanwhile given by

22 $${\hat{H}}_{I}^{(2)}\to -\,\frac{{e}^{2}{a}^{2}{\mathscr{T}}^{\prime} }{8{\hslash }^{2}}\frac{{A}_{1}{A}_{2}^{\ast }}{4}{e}^{{\rm{i}}(qx-\omega t)}{\hat{\sigma }}_{x},$$

at both $\overrightarrow{K}$ and $-\,\overrightarrow{K}$, where we have taken $\overrightarrow{A}$ to be in the form $\overrightarrow{A}(\overrightarrow{r},t)=\mathrm{(1}/\mathrm{2)}{\sum }_{m=\mathrm{1,2}}\,[{A}_{m}\hat{x}\,{e}^{{\rm{i}}({q}_{m}x-{\omega }_{m}t)}+{\rm{c}}{\rm{.c}}\mathrm{.]}$, where *q*_*m*_ is the in-plane component of the momentum and only kept the terms that give rise to a perturbation at *ω* = *ω*_1_ − *ω*_2_ and *q* = *q*_1_ − *q*_2_ (DFG). For ${\hat{H}}_{0}$ we find that the single-particle eigenenergies ${ {\mathcal E} }_{s}=s{v}_{F}\hslash k$ and eigenstates are

23 $${\psi }_{\overrightarrow{k}s}=\frac{1}{\sqrt{2\xi }}\,{e}^{{\rm{i}}\overrightarrow{k}\cdot \overrightarrow{r}}{\chi }_{s},\,{\rm{with}}\,{\chi }_{s}=(\begin{array}{c}1\\ s{e}^{i{\theta }_{\overrightarrow{k}}}\end{array})\,{\rm{at}}\,\overrightarrow{K},\,{\rm{and}}\,{\chi }_{s}=(\begin{array}{c}-s{e}^{i{\theta }_{\overrightarrow{k}}}\\ 1\end{array})\,{\rm{at}}-\,\overrightarrow{K}.$$

Here *s* = ±1 is the band index, *ξ* is the area of the graphene sheet, and ${\theta }_{\overrightarrow{k}}$ is the polar angle of $\overrightarrow{k}$.

Similarly, the spinor forms of the current densities can be found as

24 $${\hat{\overrightarrow{j}}}_{0}(\overrightarrow{r})\to -\,e{v}_{F}\hat{\overrightarrow{\sigma }}\,{\rm{at}}\,\overrightarrow{K},\,{\rm{and}}\,e{v}_{F}{\hat{\overrightarrow{\sigma }}}^{\ast }\,{\rm{at}}-\,\overrightarrow{K},$$

25 $${\hat{j}}_{x}^{(1)}\,\to \,\frac{{e}^{2}{a}^{2}{\mathscr{T}}\,^{\prime} }{4{\hslash }^{2}}\,A(\overrightarrow{r},t){\hat{\sigma }}_{x}\,{\rm{at}}\,\pm \,\overrightarrow{K},\,{\rm{and}}\,{\hat{j}}_{x}^{(2)}\,\to \,\pm \,\frac{{e}^{3}{a}^{2}{v}_{F}}{32\hslash }\,{A}_{1}{A}_{2}^{\ast }{e}^{{\rm{i}}(qx-\omega t)}{\hat{\sigma }}_{x}\,\mbox{''}+\mbox{''}{\rm{at}}\,\overrightarrow{K}\,{\rm{and}}\,\mbox{''}-\mbox{''}{\rm{at}}-\,\overrightarrow{K}.$$

It should be noted that in addition to the “typical” contribution to the current density, Eq. (24), which is associated with the Bloch momentum, there is also the “diamagnetic” term of ${\hat{j}}_{x}^{\mathrm{(1)}}$ in Eq. (25). We will see in the following that this term, and in fact a higher correction to this term of order $\overrightarrow{k}$, cancels the term that could otherwise cause a divergence in the linear conductivity.

### Density matrix method

The time evolution of the density matrix under the interaction with EM fields described by ${\hat{H}}_{I}$ is given by ${\rm{d}}\hat{\rho }/dt=(i/\hslash )[\hat{\rho },{\hat{H}}_{0}+{\hat{H}}_{I}]$. From perturbation theory, the matrix elements ${\rho }_{nm}\equiv \langle n|\hat{\rho }|m\rangle$ of the *i* th order perturbation of $\hat{\rho }$ satisfy

26 $$\frac{{\rm{d}}}{{\rm{d}}t}{\rho }_{nm}^{(i)}=-\,{\rm{i}}\,{\omega }_{nm}\,{\rho }_{nm}^{(i)}-\frac{{\rm{i}}}{\hslash }{[{\hat{H}}_{I},{\hat{\rho }}^{(i-\mathrm{1)}}]}_{nm}-\gamma \,{\rho }_{nm}^{(i)},\,(i\ge \mathrm{1)}$$

in which *n*, *m* are dummy indices denoting both the band index *s* and wavevector $\overrightarrow{k}$, ${\omega }_{nm}=({ {\mathcal E} }_{n}-{ {\mathcal E} }_{m})/\hslash$, and *γ* is a phenomenological dissipation term introduced universally for all matrix elements. We take the density matrix in absence of fields to be the Fermi distribution *f*_*n*_ with Fermi energy $\hslash {\omega }_{F}$ at zero temperature, ${\rho }_{nm}^{(0)}={\rm{\Theta }}(\hslash {\omega }_{F}-{ {\mathcal E} }_{n}){\delta }_{mn}$, where Θ is the Heaviside step function. A solution to Eq. (26) can be written as

27 $${\rho }_{nm}^{(i)}(t)=-\,\frac{{\rm{i}}}{\hslash }{\int }_{-\infty }^{t}\,{\rm{d}}t^{\prime} {[{\hat{H}}_{I}(t^{\prime} ),{\hat{\rho }}^{(i-\mathrm{1)}}(t^{\prime} )]}_{nm}{e}^{-({\rm{i}}{\omega }_{nm}+\gamma )(t-t^{\prime} )}.$$

### Linear conductivity

In previous works calculating graphene conductivities using the vector potential^8,9,11^, the authors typically replace $\hat{\overrightarrow{p}}$ by $\hat{\overrightarrow{p}}+e\overrightarrow{A}$ in the Dirac Hamiltonian. However, the linear current thus calculated has a term that diverges when the integration limit of the electronic momenta is taken to be infinity. This issue was fixed in^11^ by adding an artificial quadratic term to the Dirac Hamiltonian. In this section we show that this problematic term is actually canceled by a term in Eq. (7) in the Results section, thus no artificial term needs to be introduced to regularize the calculation.

To begin with we consider the current response to an in-plane electric field described by a vector potential $\overrightarrow{A}(\overrightarrow{r},t)=(1/2)A\hat{x}{e}^{{\rm{i}}(\overrightarrow{q}\cdot \overrightarrow{r}-\omega t)}+{\rm{c}}.{\rm{c}}.,$ where $\overrightarrow{q}=q\hat{x}$. The current generated at $\overrightarrow{q}$ and *ω* is calculated through the expectation value of ${\hat{j}}_{x}(\overrightarrow{q})=2/\xi \,{e}^{-iqx}\,{\hat{j}}_{x}(\overrightarrow{r}),$ applied to the density matrix

28 $$\langle {\hat{j}}_{x}(\overrightarrow{q})\rangle ={\rm{Tr}}(\hat{\rho }\,{\hat{j}}_{x}(\overrightarrow{q}))\approx \sum _{n,m}\,{\rho }_{nm}^{\mathrm{(1)}}\,{j}_{x}^{\mathrm{(0)}}{(\overrightarrow{q})}_{mn}+{\rho }_{nm}^{\mathrm{(0)}}\,{j}_{x}^{\mathrm{(1)}}{(\overrightarrow{q})}_{mn},$$

where the superscripts (0), (1),… denote the order of $\overrightarrow{A}$ included in the terms. According to Eq. (27), the first-order density matrix is given by

29 $${\rho }_{nm}^{(1)}(t)=\frac{eA{v}_{F}}{2\hslash }\frac{{f}_{m}-{f}_{n}}{\omega -{\omega }_{nm}+i\gamma }\langle n|{\hat{\sigma }}_{x}{e}^{iqx}|m\rangle {e}^{-i\omega t}.$$

The matrix elements of the current density operators can be obtained by using Eqs (23–25). Now we can substitute these results into Eq. (28). We then replace the summation on states by an integral over Bloch momenta $\overrightarrow{k}$, introducing an upper bound on the range of integration $k < {k}_{c}$ (which approximately captures the edge of the Brillouin zone). The first term of Eq. (28) becomes

30 $$\sum _{n,m}\,{\rho }_{nm}^{\mathrm{(1)}}\,{j}_{x}^{\mathrm{(0)}}{(\overrightarrow{q})}_{mn}=\frac{{e}^{2}A{v}_{F}}{8\pi \hslash }{e}^{-{\rm{i}}\omega t}\{2({k}_{c}-{k}_{F})+\frac{\omega }{2{v}_{F}}[\mathrm{ln}\,\frac{\omega +2{\omega }_{F}}{|\omega -2{\omega }_{F}|}+{\rm{i}}\pi \,{\rm{\Theta }}(\omega -2{\omega }_{F})]\},$$

where *k*_*F*_ is the Fermi wavevector, and the second term gives zero. If *k*_*c*_ is extended to infinity like in a free-electron gas, Eq. (30) will yield a divergent linear current, as discussed by Wang^11^. We show next how this strong dependence on *k*_*c*_ is cancelled by the lowest order non-zero contribution to the second term in Eq. (28).

We expand the linear current density operator Eq. (6) to first order of $\overrightarrow{k}$, and obtain an additional term to ${\hat{j}}_{x}^{\mathrm{(1)}}$ in Eq. (25) which we label as ${\hat{j}}_{x}^{\mathrm{(1})^{\prime} }$:

31 $${\hat{j}}_{x}^{(1)^{\prime} }=\frac{{e}^{2}{a}^{2}}{4{\hslash }^{2}}{v}_{F}A(\overrightarrow{r},t)(\pm {\hat{\sigma }}_{x}{\hat{p}}_{x}+\frac{1}{3}{\hat{\sigma }}_{y}{\hat{p}}_{y}),$$

where “+” or “−” sign corresponds to the Dirac point $\overrightarrow{K}$ or $-\overrightarrow{K}$. Now the second term in Eq. (28) has an additional term which gives a finite contribution:

32 $$\sum _{n,m}\,{\rho }_{nm}^{\mathrm{(0)}}\,{j}_{x}^{\mathrm{(1})^{\prime} }{(\overrightarrow{q})}_{mn}=-\,\frac{{e}^{2}A{v}_{F}}{8\pi \hslash }{e}^{-{\rm{i}}\omega t}\frac{{a}^{2}({k}_{c}^{3}-{k}_{F}^{3})}{9}.$$

We note ${k}_{F}\ll {k}_{c}$ and ${k}_{F}\gg {a}^{2}{k}_{F}^{3}$, thus Eq. (32) cancels with the *k*_*c*_ term in Eq. (30) at ${k}_{c}=3\sqrt{2}/a\approx |\overrightarrow{K}|$, the edge of the Brillouin zone^27^. An exact calculation beyond the Dirac cone approximation would also result in the same qualitative cancellation and a small correction depending on the details of the entire band structure.

Eliminating *A* by $\overrightarrow{E}=-\,\partial \overrightarrow{A}/\partial t$ in the expression of $\langle {\hat{j}}_{x}(\overrightarrow{q})\rangle$, using the definition $\overrightarrow{j}={\sigma }^{(1)}\overrightarrow{E}$ where ${\sigma }^{(n)}$ denotes the *n* th order conductivity, and multiplying the valley and spin degeneracy factor of 4, we finally reach

33 $${\sigma }^{\mathrm{(1)}}(\omega )=\frac{{e}^{2}}{\hslash }[\frac{1}{4}\,{\rm{\Theta }}\,(\omega -2{\omega }_{F})+\frac{{\rm{i}}}{\pi }(\frac{{\omega }_{F}}{\omega }+\frac{1}{4}\,\mathrm{ln}\,\frac{|\omega -2{\omega }_{F}|}{\omega +2{\omega }_{F}})],$$

which is in agreement with the result derived by various other theoretical approaches, *e.g*. using a scalar potential. We have seen both ${\hat{\overrightarrow{j}}}_{0}$ and ${\hat{\overrightarrow{j}}}^{\mathrm{(1)}}$ (Eqs (5) and (6) in Results) play an important role in obtaining the correct linear conductivity; they are actually analogous to the paramagnetic and diamagnetic parts of current respectively in the case of free electrons coupled to a vector potential. However the replacement $\hat{\overrightarrow{p}}\to \hat{\overrightarrow{p}}+e\overrightarrow{A}$ in the Dirac Hamiltonian would only yield the “paramagnetic” part and therefore give incorrect result for the linear current. Thus the Dirac Hamiltonian is insufficient when using a vector potential and one has to start with the original tight-binding Hamiltonian. We note that the issue with using a vector potential with the Dirac Hamiltonian has been known before, as was pointed out in the works studying optical sum rules^27,28^.

### Nonlinear conductivity

We consider the nonlinear current in response to the EM fields described by $\overrightarrow{A}(\overrightarrow{r},t)=(1/2){\sum }_{m=1,2}\,[{A}_{m}\hat{x}\,{e}^{i({q}_{m}x-{\omega }_{m}t)}+{\rm{c}}{\rm{.c}}{\rm{.}}]$, at difference frequency *ω*_3_ = *ω*_1_ − *ω*_2_ and wavevector *q*_3_ = *q*_1_ − *q*_2_. Similar to Eq. (28) the nonlinear current can be calculated using the density matrix as

34 $$\langle {\hat{j}}_{x}({\overrightarrow{q}}_{3})\rangle ={\rm{Tr}}(\hat{\rho }\,{\hat{j}}_{x}({\overrightarrow{q}}_{3}))\approx \sum _{n,m}\,{\rho }_{nm}^{\mathrm{(0)}}\,{j}_{x}^{\mathrm{(2)}}{({\overrightarrow{q}}_{3})}_{mn}+{\rho }_{nm}^{\mathrm{(1)}}\,{j}_{x}^{\mathrm{(1)}}{({\overrightarrow{q}}_{3})}_{mn}+{\rho }_{nm}^{\mathrm{(2)}}\,{j}_{x}^{\mathrm{(0)}}{({\overrightarrow{q}}_{3})}_{mn},$$

Using Eqs (23) and (25), we find the first term in Eq. (34) vanishes due to the angular integral. For the second term in the summation, one notes using Eqs (23), (25) and (29), ${j}_{x}^{\mathrm{(1)}}{({\overrightarrow{q}}_{3})}_{mn}$ flips sign at $\overrightarrow{K}$ and $-\,\overrightarrow{K}$ whilst ${\rho }_{nm}^{\mathrm{(1)}}$ stays the same, thus the two contributions at $\overrightarrow{K}$ and $-\,\overrightarrow{K}$ cancel. There are two distinctive contributions to the matrix element ${\rho }_{nm}^{\mathrm{(2)}}$ in the third term: there is one contribution coming from the nonlinear vector potential interaction Eq. (22), which when acting upon the equilibrium density matrix ${\hat{\rho }}^{\mathrm{(0)}}$ (see Eq. (27)) produces density matrix ${\hat{\rho }}^{\mathrm{(2)}}$ oscillating at the difference frequency:

35 $${\rho }_{nm}^{\mathrm{(2)}}(t)=-\,\frac{{e}^{2}\,{A}^{2}t}{32{\hslash }^{2}}{A}_{1}{A}_{2}^{\ast }\frac{{f}_{m}-{f}_{n}}{\omega -{\omega }_{nm}+{\rm{i}}\gamma }\langle n|{\hat{\sigma }}_{x}{e}^{{\rm{i}}qx}|m\rangle {e}^{-{\rm{i}}{\omega }_{3}t}\mathrm{.}$$

This term flips sign at $\overrightarrow{K}$ and $-\,\overrightarrow{K}$, while ${j}_{x}^{\mathrm{(0)}}{({\overrightarrow{q}}_{3})}_{mn}$ does not. Thus the two contributions at $\overrightarrow{K}$ and $-\,\overrightarrow{K}$ cancel. There is a further contribution coming from the frequency 1(2) component in the linear interaction Eq. (21)) acting upon ${\hat{\rho }}^{\mathrm{(0)}}$ through Eq. (27) to generate a first order perturbation ${\hat{\rho }}^{\mathrm{(1)}}$, and then the frequency 2(1) component in ${\hat{H}}_{I}^{\mathrm{(1)}}$ acting upon ${\hat{\rho }}^{\mathrm{(1)}}$, and generating a nonlinear perturbation ${\hat{\rho }}^{\mathrm{(2)}}$ at the difference frequency. Using Eqs (21), (27), and (29) one gets

36 $$\begin{array}{c}{\rho }_{nm}^{\mathrm{(2)}}(t)=\frac{{e}^{2}{v}_{F}^{2}{A}_{1}{A}_{2}^{\ast }}{4{\hslash }^{2}}{e}^{-{\rm{i}}{\omega }_{3}t}\frac{1}{{\omega }_{3}-{\omega }_{nm}+{\rm{i}}\gamma }\times \sum _{l}\,(\frac{{f}_{m}-{f}_{l}}{-{\omega }_{2}-{\omega }_{lm}+{\rm{i}}\gamma }-\frac{{f}_{l}-{f}_{n}}{{\omega }_{1}-{\omega }_{nl}+{\rm{i}}\gamma })\\ \,\,\,\times \langle n|{\hat{\sigma }}_{x}{e}^{{\rm{i}}{q}_{1}x}|l\rangle \langle l|{\hat{\sigma }}_{x}{e}^{-{\rm{i}}{q}_{2}x}|m\rangle +({q}_{1}\leftrightarrow -{q}_{2},{\omega }_{1}\leftrightarrow -{\omega }_{2})\mathrm{.}\end{array}$$

One can then carry out the summation in Eq. (34). All band combinations need to be considered $({s}_{n},{s}_{m},{s}_{l}=\pm \,1)$. The summation can be transformed to an integral, which in general needs to be evaluated numerically. However we can also expand the kernel in terms of *q*_3_, and extract the leading order contributions. Under the experimental conditions of both^7^ and^14^: (i.e. ${\omega }_{3} < {\omega }_{F}\ll {\omega }_{1}\approx {\omega }_{2}$), we obtain

37 $$\langle {\hat{j}}_{x}^{\mathrm{(2)}}(\overrightarrow{q})\rangle =-\,\frac{{e}^{3}{v}_{F}^{2}{A}_{1}{A}_{2}^{\ast }}{2\pi {\hslash }^{2}}(\frac{{q}_{3}}{{\omega }_{3}})\frac{{\omega }_{F}^{4}}{({\omega }_{3}^{2}-4{\omega }_{F}^{2}){\omega }_{2}^{2}};$$

the current contribution at $-\,\overrightarrow{K}$ is the same. Thus, changing the potentials to electric fields, using the definition $\langle {\hat{j}}_{x}^{\mathrm{(2)}}({\overrightarrow{q}}_{3})\rangle ={\sigma }^{\mathrm{(2)}}{E}_{1}{E}_{2}^{\ast }$, and introducing the spin and valley degeneracy factor of 4, we finally reach Eq. (8) in the Results.

### Transmission and reflection coefficients

To make this contribution self-contained, in this subsection we briefly review the theoretical model developed in the Supplementary Information of Constant^14^ to describe the input and nonlinear electric fields in the experiment. This model assumes a linear frequency dependence of the second order nonlinear graphene conductivity. However, we have tested that this assumption does not significantly modify the result, since the differential reflection signal in the model is predominantly determined by absorption of the difference frequency field, and hence by the magnitude of the conductivity at the difference frequency. Throughout this paper we follow Constant^14^ and take a plasmon linewidth of order ~10 THz.

The convention to define the field polarizations and beam angles is illustrated in Fig. 1. In general, the transmission coefficients can be found by imposing boundary conditions at the graphene interface (continuity of the normal electric displacement and tangential electric field). For the transmission coefficient of field *i* (*i* = 1, 2),

38 $${t}_{i}=\frac{2\,\sin \,{\theta }_{i}}{{n}_{i}\,\sin \,{\theta }_{i}+\,\sin \,{\varphi }_{i}}-(\frac{{\rho }_{is}}{{\varepsilon }_{0}{E}_{Ii}})\frac{\sin \,{\theta }_{i}}{{n}_{i}\,\cos \,{\varphi }_{i}({n}_{i}\,\sin \,{\theta }_{i}+\,\sin \,{\varphi }_{i})},$$

where *E*_*Ii*_ are the incident field amplitudes, ${n}_{i}=n({\omega }_{i})$ denotes the index of refraction of the substrate at the field frequency, and ${\rho }_{is}=\rho ({\omega }_{i},{q}_{i})$ is the graphene surface charge density at frequency ${\omega }_{i}$ and in-plane wavevector ${q}_{i}=({\omega }_{i}/c)\,\cos \,{\theta }_{i}$. The reflection coefficients *r*_*i*_ can be obtained via the relation ${r}_{i}+(\sin \,{\varphi }_{i}/\,\sin \,{\theta }_{i}){t}_{i}=1$. As in Constant^14^, we take the substrate model given by Luxmoore^29^:

39 $${n}^{2}(\omega )={\varepsilon }_{\infty }+\sum _{j=1}^{3}\,\frac{{f}_{j}\,{\omega }_{TO,j}^{2}}{{\omega }_{TO,j}^{2}-{\omega }^{2}-i\omega {\gamma }_{TO,j}}.$$

The high-frequency dielectric constant ${\varepsilon }_{\infty }=2.4$, and ${\omega }_{TO}=2\pi \times (13.44,23.75,33.84)$ THz, ${\gamma }_{TO}=2\pi \times (0.80,1.27,1.27)$ THz, and $f=(0.7514,0.1503,0.6011)$ are the frequencies, damping rates, and oscillator weights of the three transverse optical phonon modes respectively. In practice, Eq. (39) is only relevant at low difference frequency of *ω*_3_ = *ω*_1_ − *ω*_2_, whereas for the high pump and probe frequencies *ω*_1,2_ the index of refraction is nearly a constant: $n\approx \sqrt{{\varepsilon }_{\infty }}$. ${\rho }_{is}$ can be related to the current density *j* in the graphene layer via the continuity equation, in the Fourier space yielding: ${\rho }_{is}({\omega }_{i},{q}_{i})=({q}_{i}/{\omega }_{i}){j}_{x}({\omega }_{i},{q}_{i})$. When depletion can be ignored, as is the case in Constant^14^, the current densities at these frequencies can be approximated by their linear response results:

40 $${j}_{x}({\omega }_{i},{q}_{i})={\sigma }^{(1)}({\omega }_{i}){E}_{x}({\omega }_{i},{q}_{i}).$$

Here, ${E}_{x}({\omega }_{i},{q}_{i})={t}_{i}{E}_{Ii}\,\sin \,{\varphi }_{i}$ are the total parallel fields in the graphene layer.

The field generated at the difference frequency *ω*_3_ = *ω*_1_ − *ω*_2_ can be found similarly by using the boundary conditions at the graphene interface. We note in Fig. 1 now there is no incident field, but only reflected and transmitted fields ${\overrightarrow{E}}_{3R}$ and ${\overrightarrow{E}}_{3T}$ respectively. Using the boundary conditions for the electric fields and displacements at the graphene layer, we have:

41 $${E}_{3R}\,\sin \,{\theta }_{3}+{E}_{3T}\,\sin \,{\varphi }_{3}=\mathrm{0,}$$

42 $${E}_{3R}\,\cos \,{\theta }_{3}-{n}^{2}({\omega }_{3}){E}_{3T}\,\cos \,{\varphi }_{3}={\rho }_{3s}/{\varepsilon }_{0}.$$

The surface charge density ${\rho }_{3s}$ can be expressed in terms of the current density ${j}_{x}({\omega }_{3},{q}_{3})$. We note now a source term *j*_*s*_ is included in *j*_*x*_ depending on the origin of DFG; this term describes the generated field independent from the graphene’s linear response to the field at the frequency: ${j}_{x}({\omega }_{3},{q}_{3})={\sigma }^{(1)}({\omega }_{3}){E}_{3x}+{j}_{s}({\omega }_{3},{q}_{3})$. *j*_*s*_ can arise from different nonlinear processes, e.g. in Constant^14^ this is taken as a result of graphene nonlinearity, while in this contribution this originates from the Seebeck effect, and can then induce a plasmon field. Using Eqs (41) and (42) and the expression of *j*_*x*_, one can then solve for *E*_3*x*_.

## Data Availability

All data created during this research are openly available from the University of Exeter’s institutional repository located at https://ore.exeter.ac.uk/repository/handle/10871/35749.
